# Antibacterial Activity of Bacterial Cellulose Loaded with Bacitracin and Amoxicillin: In Vitro Studies

**DOI:** 10.3390/molecules25184069

**Published:** 2020-09-06

**Authors:** Georgiana-Mădălina Lemnaru (Popa), Roxana Doina Truşcă, Cornelia-Ioana Ilie, Roxana Elena Țiplea, Denisa Ficai, Ovidiu Oprea, Anicuța Stoica-Guzun, Anton Ficai, Lia-Mara Dițu

**Affiliations:** 1 National Centre for Micro and Nanomaterials and National Centre for Food Safety, Faculty of Applied Chemistry and Materials Science, University POLITEHNICA of Bucharest, Splaiul Independentei 313, 060042 Bucharest, Romania; lemnaru_madalina92@yahoo.com (G.-M.L.); truscaroxana@yahoo.com (R.D.T.); cornelia.ilie18@gmail.com (C.-I.I.); roxanatiplea@yahoo.com (R.E.Ț.); denisa.ficai@upb.ro (D.F.); astoica.upb@gmail.com (A.S.-G.); 2 Academy of Romanian Scientists, 3 Ilfov Street, 050045 Bucharest, Romania; 3 Faculty of Biology, University of Bucharest, 1-3 Aleea Portocalelor, 060101 Bucharest, Romania; lia-mara.ditu@bio.unibuc.ro or

**Keywords:** bacterial cellulose, skin wounds, antimicrobial effect, drug delivery, dressings

## Abstract

The use of bacterial cellulose (BC) in skin wound treatment is very attractive due to its unique characteristics. These dressings’ wet environment is an important feature that ensures efficient healing. In order to enhance the antimicrobial performances, bacterial-cellulose dressings were loaded with amoxicillin and bacitracin as antibacterial agents. Infrared characterization and thermal analysis confirmed bacterial-cellulose binding to the drug. Hydration capacity showed good hydrophilicity, an efficient dressing’s property. The results confirmed the drugs’ presence in the bacterial-cellulose dressing’s structure as well as the antimicrobial efficiency against *Staphylococcus aureus* and *Escherichia coli*. The antimicrobial assessments were evaluated by contacting these dressings with the above-mentioned bacterial strains and evaluating the growth inhibition of these microorganisms.

## 1. Introduction

An ideal wound dressing should facilitate a suitable healing environment, allow rapid tissue regeneration, reduce pain, and prevent infection during the healing process. The wound must be hydrated, while the excess of exudates and toxins must be removed and oxygen penetration must take place to accelerate healing [1,2]. Bacterial cellulose (BC) is an extracellular polysaccharide produced by several bacteria, in the form of a membrane (hydrogel with ~99% water) at the air/medium culture interface. Biosynthesis of BC is achieved by the polymerization of uracil-diphosphate glucose (UDP-glucose) into α-1, 4-glucan chains by the involvement of various enzymes and then microfibers are formed in a template-assisted manner; bacterial cells’ distribution within the BC matrix represents the porogenic template, and upon their removal, a porous network of BC fibers results [3]. Bacterial cellulose (BC) is chemically identical to plant cellulose (PC) but possesses different macromolecular structure and physical properties that give it special features, which are not encountered in cellulose obtained from plants. For example, BC has a higher crystallinity than plant cellulose while the fiber diameter is smaller, assuring a higher surface/volume ratio. It is also free of lignin, hemicelluloses, and pectin, which are found together with cellulose in the plant structure. It also has remarkable mechanical properties and high-water holding capacity. BC is a network structure that could be modified in fermentation medium using different additives and various fermentation conditions [4]. Three free hydroxyl groups are present on each glucose ring of BC, so that the microfibers self-assemble into a bundle of fibers with a diameter of 10–100 nm due to the large number of hydrogen bonds, and then form a three-dimensional network of nanofibers with a large number of pores that can host both biological active substances, minerals, as well as cells [5]. Additionally, due to these hydroxyl groups, strong interactions can appear with the hydrophilic biological active agents (especially those bearing OH, COOH, NH_2_ groups), which can be released from the cellulose-based supports in a controlled manner [6,7]. This network architecture confers to BC tremendous possibilities to be used in various fields but especially in the medical field as a skin wound and burn cure, artificial skin, artificial blood vessels, dental implants, scaffolds for tissue engineering, and drug delivery systems, with the porous structure ensuring an efficient cell attachment and high capacity of exudate adsorption [8]. It is characterized by a good permeability and resistance to degradation, good mechanical properties, high purity and high water absorption capacity, remarkable biocompatibility, and high porosity [1,9,10,11,12,13]. Not only BC but also BC composites are considered promising wound dressing materials [14].

The particular nano-fibrillar structure of BC is a suitable macromolecular support for the adsorption of drugs and therefore for the development of specific controlled release systems, including wound dressing and transdermal drug delivery systems [15]. The BC membrane’s ability to facilitate active substances’ release for percutaneous administration has been demonstrated [12]. It is also a neutral electrostatic material, which allows both negative and positive charges of bioactive compounds. In addition, high water retention creates a moist wound healing environment and allows exudate adsorption, thus fast healing [1]. Even if BC has an excellent biocompatibility recommending it for wound healing, it has no bacterial activity, being one of its drawbacks for biomedical applications. For this reason, many organic substances (e.g., antibiotics, antimicrobial peptides, cationic antibacterial agents) or inorganic nanoparticles were loaded in the BC network in order to confer antibacterial properties. The most common examples are BC-Ag and BC-ZnO nanocomposites [2,16]. Composites of BC-chitosan were also tested as biomaterials for a wound dressing [17]. A recent attempt was done by immobilization of *Bacillus subtilis* (BS) cells on BC matrix in order to obtain a material with a wound-healing effect [18]. Recent studies regarding the BC dressing use for burn treatment have demonstrated that BC containing antimicrobial agents could be a better choice in comparison with sulfadiazine cream [19,20].

Our study aimed to load bacterial cellulose membranes with antibiotics (bacitracin and amoxicillin) at different concentrations for increased antibacterial activity of these membranes. The bacterial cellulose membranes’ functionality was demonstrated by evaluating the antibacterial effect on *Escherichia coli* and *Staphylococcus aureus*. Currently, amoxicillin has been loaded in cellulose aerogel (cellulose of vegetal origin) [21], but no paper has reported the bacitracin loading in cellulose and especially in bacterial cellulose.

## 2. Results and Discussion

The antibiotic-loaded BC membranes were characterized by adequate physical-chemical tools but also by evaluating the water uptake and antimicrobial performances.

### 2.1. Scanning Electron Microscopy (SEM)

Scanning electron microscopy (SEM) is particularly useful for analyzing material morphology. All samples were analyzed in cross-section as well as on the surface.

**Figure 1:**
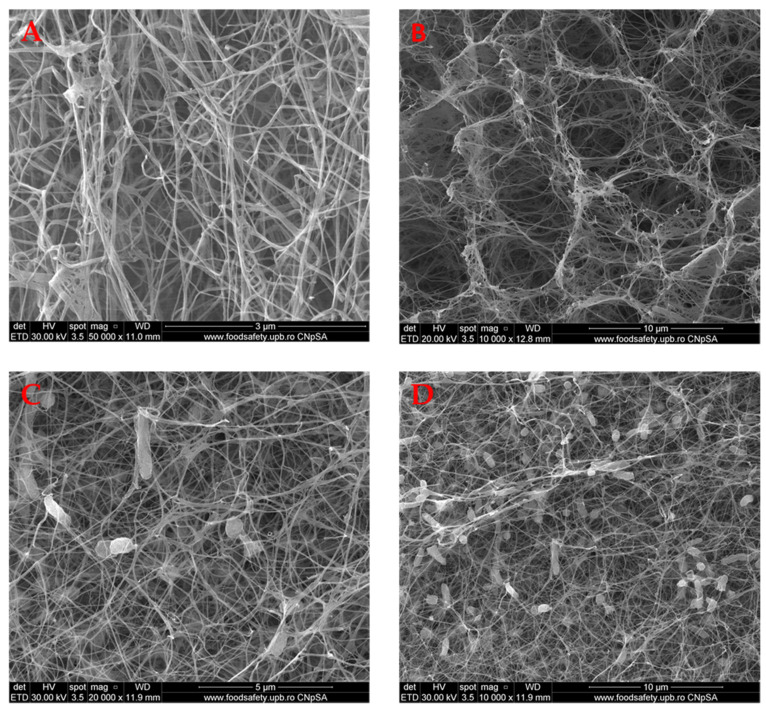
BC control SEM images, showing the fiber morphology on the surface (**A** 50,000×; **B** 10,000×) and in cross-section (**C** 20,000×; **D** 10,000×).

The SEM images presented in Figure 1 show the BC structure. It can be concluded that this is a homogeneous tridimensional structured material, with a nano- and microfibrils network of cellulose, similar to those reported in the literature [22]. It is important to mention that the wall of the pores is, in fact, nanofibrillar with many pores developed between them. This very porous structure is important and can explain some properties of BC, namely the high-water uptake, ability to absorb the exudates and toxins, but also to adsorb and release the biological active agents. It is very important to mention that the morphology is totally different compared with the cellulose aerogels’ morphology loaded or not with amoxicillin, as reported by Ye et al. [21], as the bacterial cellulose fibrillar structure is not characteristic even if, in both cases, the porosity is very high.

### 2.2. FT-IR Analysis

To verify whether BC retained its structure during the loading and lyophilization processes as well as to check the impurities from bacterial cellulose, FT-IR spectroscopy analyses were performed.

**Figure 2:**
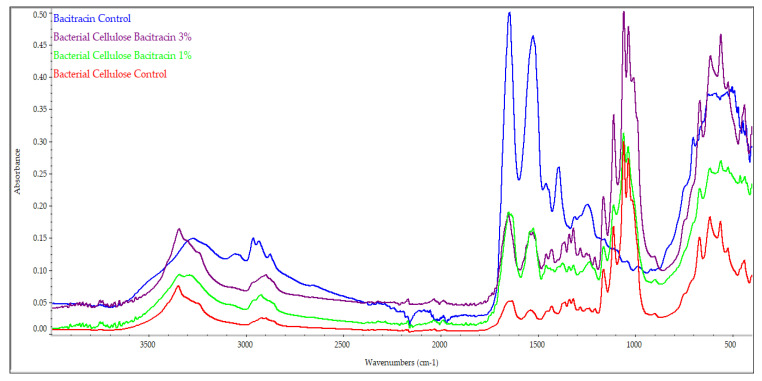
FTIR spectra of bacitracin control, BC-bacitracin 1%, BC-bacitracin 3%, and BC control.

Figure 2 shows the BC control spectra, BC-bacitracin 1%, BC-bacitracin 3%, and bacitracin control. FT-IR BC control analysis showed its characteristic chemical bonds. It can be seen that at 3346 cm^−1^, there is a stretching vibration of OH groups, and at 2896 cm^−1^, a stretching vibration of the CH_2_ and CH group. The crystallization water HOH binding vibration is identified at 1647 cm^−1^, and at 1280 cm^−1^, the OH deformation vibration can be seen. The peak at 1205 cm^−1^ is attributed to the CH deformation vibration. The asymmetrical stretch vibrations of the C-C are identified at 1160 cm^−1^ [23,24]. The band from 1156 cm^−1^ is assigned to the asymmetric C-O-C stretching vibrations. The peaks from 1308 and 1455 cm^−1^ are correlated to the CH_2_ symmetric bending and CH_2_ deformation, respectively [25]. The bacterial cells’ specific peaks, for instance, bands assigned to lipids and proteins, can be considered for the evaluation and even the semiquantitative determination of the presence of impurities derived from bacterial cells. Based on the study published by Fuller et al. [26], especially reporting the intensities of the peaks from 1160 and 1650 cm^−1^, it can be concluded that some cells are present (also visible in the SEM images), but the content of the whole cells does not reach 2%. Related to the literature, the bacitracin spectrum showed several specific adsorption bands. The absorption peak from 3270 cm^−1^ is derived from the stretching vibrations of the –OH and/or –NH groups. The absorption peaks from 2961 and 1522 cm^−1^ are correlated with the C-H and C-C stretching vibrations. Additionally, the C-C aromatic stretching vibration is represented by the absorption peak from 1643 cm^−1^, while the C-O and C-S stretching vibrations are represented by the absorption peaks from 1100 and 700 cm^−1^ [27,28]. It is important to mention that interactions between the support and the active agent appear and this is why the band between 1600 and 1700 cm^−1^ is shifting to a higher wavenumber. For instance, in BC, in the above-mentioned range, there are two peaks centered at 1631 (stronger) and 1647 cm^−1^ while in the sample loaded with 1% bacitracin along with a shift to 1636 and 1648 cm^−1^, the intensity of the last peak became stronger. In the sample loaded with 3% of bacitracin, the peak from ~1630 cm^−1^ became just a shoulder and only the peak from 1650 cm^−1^ can be easily observed. Similar discussions can be made on all the bands associated with the groups developing support–active agent interactions. The most important peaks of BC, bacitracin and the BC-bacitracin samples (from Figure 2) are summarized in Table 1.

**Figure 3:**
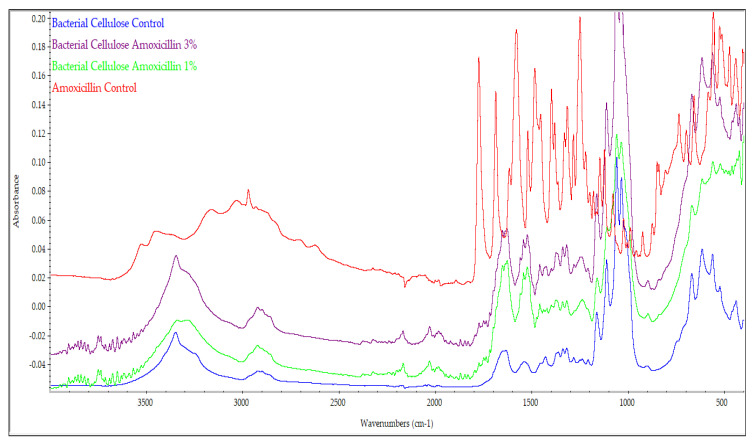
FTIR spectra for BC control, BC-amoxicillin 1%, BC-amoxicillin 3%, and amoxicillin control.

The amoxicillin-relevant IR absorption bands were identified and are reported in Table 2. These results are in accordance with the literature [29,30]. The pure amoxicillin infrared spectrum showed a strong absorption at 1772 cm^−1^, characteristic of the β-lactam ring [31]. It can also be noticed that the (Figure 3) amoxicillin addition, due to the strong interaction with the BC support, led to the splitting of some bands because, partially, amoxicillin interacts with these groups. The most evident area proving this fact is related to the area where the β-lactam ring appeared, and it can highlight several bands in the 1700–1800 cm^−1^ where BC has no characteristic peak.

### 2.3. Thermal Analysis

**Figure 4:**
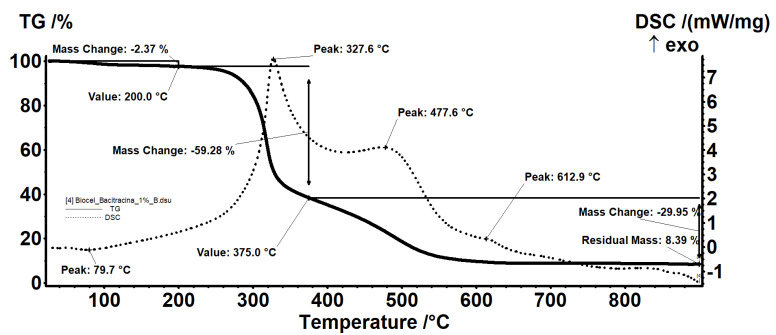
Thermal analysis of BC-bacitracin 1%.

All samples were subjected to thermal analysis. The BC-bacitracin 1% (Figure 4) sample exhibits a behavior similar to that of the control BC. In fact, all the evidence and interpretations are similar. The sample BC-bacitracin 1% is thermally stable up to 200 °C, losing 2.37% of the mass, with the process being accompanied by an endothermic effect, with a minimum at 79.7 °C. In this step, the water (solvent) used to obtain the sample is removed.

Between 200 and 375 °C, there is a mass loss of 59.28%, the process being one of oxidative degradation as indicated by the exothermic effect with a maximum at 327.1 °C that accompanies it. In the range 375–900 °C, there is a mass loss of 29.95% accompanied by two exothermic effects: A wide one, with a plateau at 477.6 °C, and a weak one visible at 612.9 °C. At this stage, the carbon mass left over from the previous process is oxidized, with the thermal effect indicating the two types of carbonic residues present: One belonging to BC, which burns at a lower temperature, and a second type of residue linked to the bacitracin skeleton, requiring a higher oxidation temperature. The residual mass is 8.39% and represents the inorganic part of the sample.

**Figure 5:**
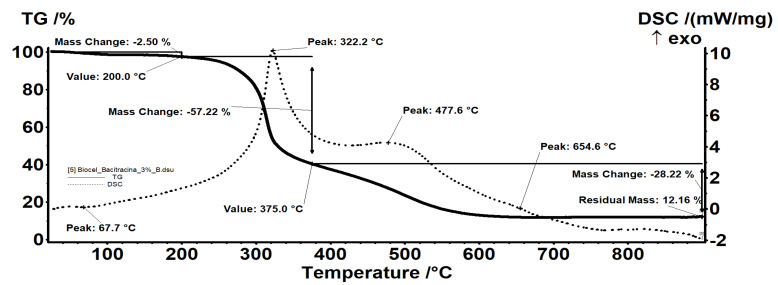
Thermal analysis of BC-bacitracin 3%.

In the case of the BC-bacitracin 3% (Figure 5) sample, it has the same effects as the BC-bacitracin 1%, only because the bacitracin concentration increases, it leads to an increase of the solvent retention. The residual mass is 12.16% and represents the inorganic part of the sample.

**Figure 6:**
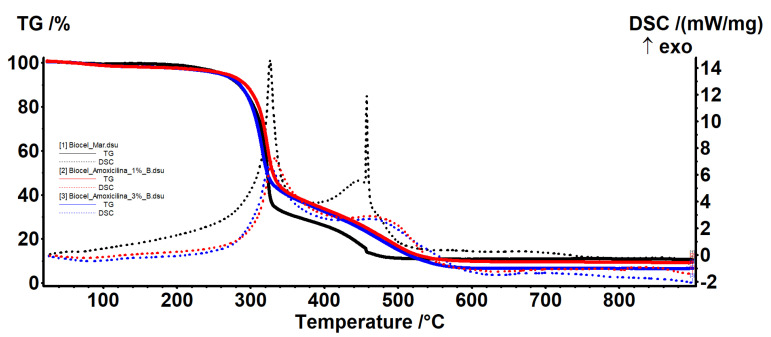
Thermal analysis of BC-amoxicillin 1% and 3%.

In the samples loaded with amoxicillin (Figure 6), the interpretation is similar. The sample is thermally stable up to 200 °C losing approximately 3% of the mass, with the process being accompanied by an endothermic effect. In this step, the water is removed. The retained water quantity is in agreement with other reports for BC [32] but is in general lower for the BC when compared with normal cellulose (~50%) [21].

The mass loss is higher than in the control sample, which indicates that at the same time as amoxicillin, additional water is retained. Between 200 and 375 °C, there is a mass loss of approximately 60% in both samples, with the process being one of oxidative degradation as indicated by the exothermic effect. In the range 375–900 °C, there is a loss of mass, determined by the burning of the carbon mass, with the process being accompanied by two exothermic effects, one wide and one noticeable. The centralization of the mass losses of all the samples is presented in Table 3.

### 2.4. Kinetic of Water Absorption

**Figure 7:**
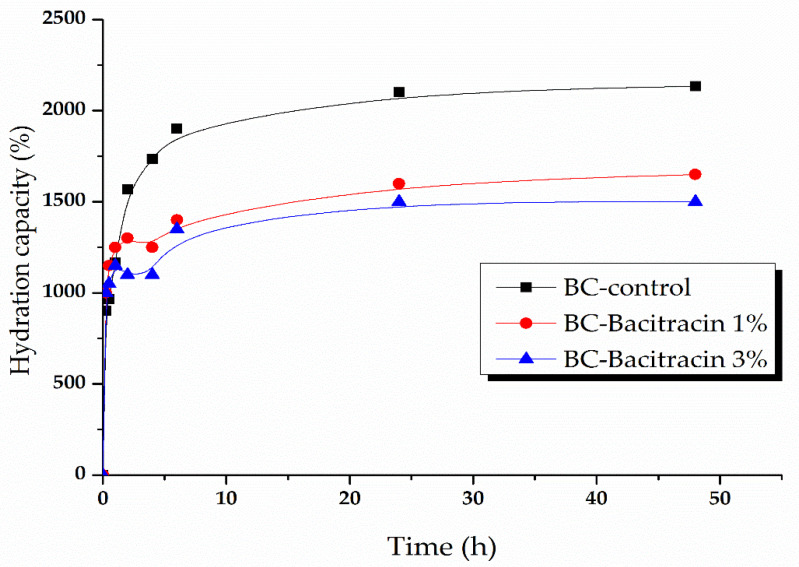
Absorption kinetics of BC samples loaded with bacitracin 1% and 3%.

**Figure 8:**
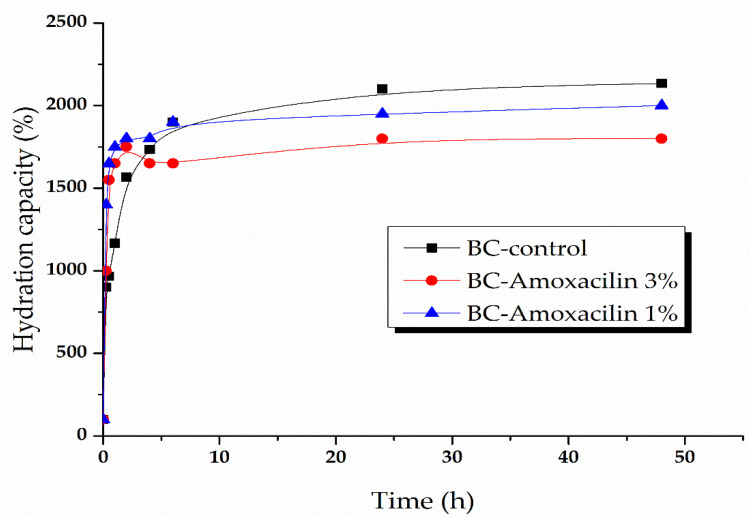
Absorption kinetics of BC samples loaded with amoxicillin 1% and 3%.

A high humidity of the material that is used as a dressing wound favors the absorption of the exudates, and the active substance’s absorption and removal allows easy and painless removal of the dressing, accelerates the wound healing process, and prevents deterioration of newly formed skin [33]. Due to its hydrophilic nature and high water absorption capacity, BC has been widely used as a wound coating material [34,35]. BC is a good drug absorption support, showing rapid and massive water absorption, which may be suitable to absorb exudate and maintain a proper humidity and oxygen permeability to ensure wound healing. The water absorption data are presented in Figure 7 and Figure 8.

The graphs presented in Figure 7 illustrate the following samples’ hydration capacity: BC-control, BC-bacitracin 1%, and BC-bacitracin 3%. The initial growth (within the first 30 min of immersion) is particularly fast for all three samples and results in a water absorption of about 1000, 1150% hydration capacity for the samples loaded with bacitracin, and ~1900% for BC. It is worth mentioning that the samples loaded with bacitracin show an abnormal evolution between 2 and 4 h most probably because the bacitracin release. The samples reach a saturation of around 2100% hydration capacity for BC-control and about 1500–1600% for BC loaded with bacitracin after ~1 day. These samples are stable for at least an additional day.

In Figure 8, the BC samples loaded with amoxicillin’s hydration capacity is presented. As in the previous case, all the samples show a rapid initial growth (within the first 30 min of immersion), reaching a percentage of 1500% water absorption for the sample with 1% amoxicillin and 1100% water absorption for the sample with 3% amoxicillin. In comparison with BC-bacitracin samples, those with amoxicillin have a lower absorption capacity than the BC-control sample. The samples reach saturation after several hours, at around 2100 for BC, 1900 for BC-amoxicillin 1%, and ~1700% for BC-amoxicillin 3%.

### 2.5. Antibacterial Assays

The logarithmic phase-delayed time determined that division and cell growth were inhibited by bacitracin. The growth rates of *Escherichia coli* and *Staphylococcus aureus* were lower in the presence of bacitracin and amoxicillin.

In this study, *Staphylococcus aureus* has higher sensitivity compared to *Escherichia coli* to different biologically active substances, a result that correlates with data from the literature [36]. The cell wall complex structure (peptidoglycan complex-lipoprotein and outer membrane) in the case of Gram-negative strains generally determines resistance to the different pharmaceutical formulations’ action [36,37].

**Figure 9:**
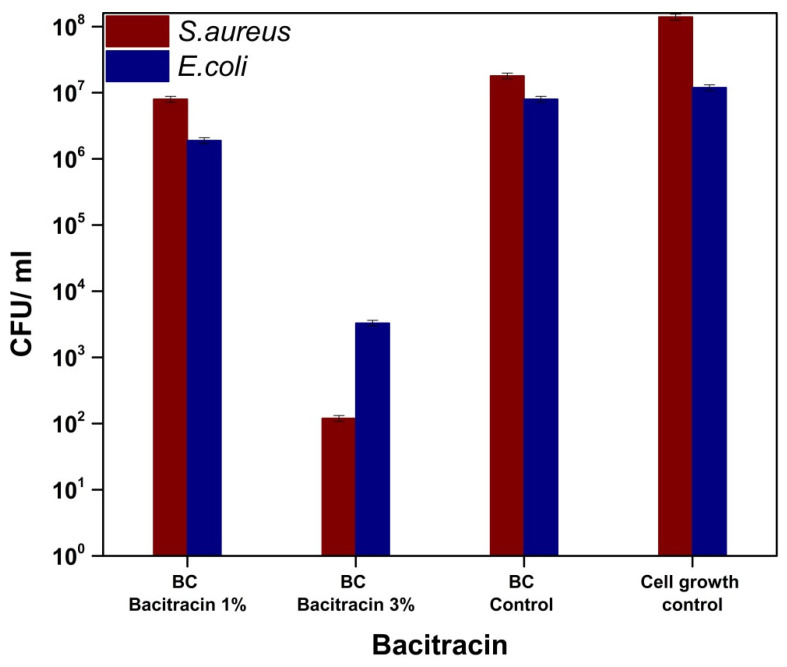
Graphical representation of CFU/mL values of *Staphylococcus aureus* ATCC 25923 and *Escherichia coli* ATCC 25922, in order to evaluate the capability of the bacterial cells to adhere on the surface of BC-bacitracin samples.

**Figure 10:**
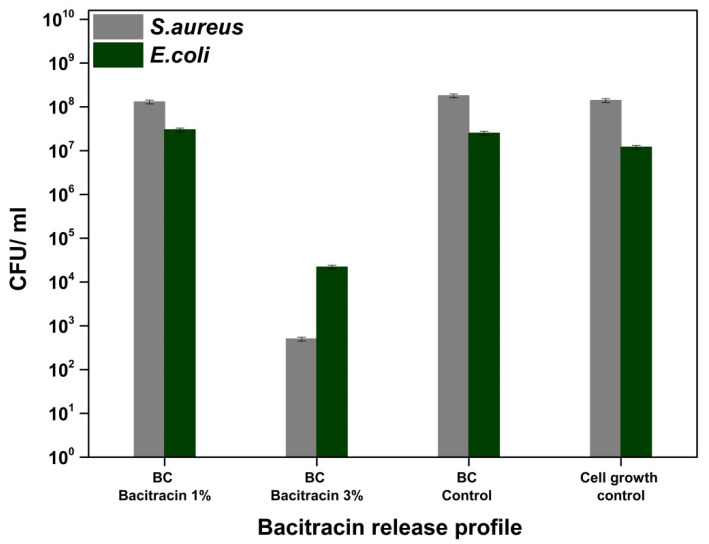
Graphical representation of CFU/mL values of *Staphylococcus aureus* ATCC 25923 and *Escherichia coli* ATCC 25922, in order to evaluate the bacitracin release from the BC-bacitracin samples into the broth media.

Bacitracin, a polypeptide with a complex structure that is derived from *Bacillus subtilis*, inhibits the synthesis of peptidoglycan, and the formation of the cell wall [38,39]. Bacitracin is used for Gram-positive bacterial infections and is administered topically [38,39,40]. It can be observed in Figure 9 a decrease of the CFU/mL values in the case of the treated materials compared to the untreated sample, respectively, to the cell growth control. Additionally, the sample loaded with 3% bacitracin resulted in a decrease of at least four logarithmic units in both strains tested, which implies the ability to inhibit the adhesion of microorganisms on the surface of materials. In general, skin lesions/injuries are attended by infections due to the presence of microorganisms [38,41,42], and the effectiveness of loaded materials for treating skin wounds is demonstrated by the release of bacitracin from their composition, implicitly by inhibiting cell multiplication in the solution (Figure 10-bacitracin release/*S. aureus* and *E. coli*) [38,39,43,44,45]. The growth rate of *Escherichia coli* and *Staphylococcus aureus* decreased as the bacitracin concentration increased [46]. So, the loading of bacterial cellulose with bacitracin induced a non-adhesive surface to both *S. aureus* and *E. coli* while, due to the release of the antibiotic, the biocidal activity can be clearly identified for the BC-bacitracin 3% and these data demonstrate that this system can be used for both preventive purposes but also for the treatment of infected wounds.

**Figure 11:**
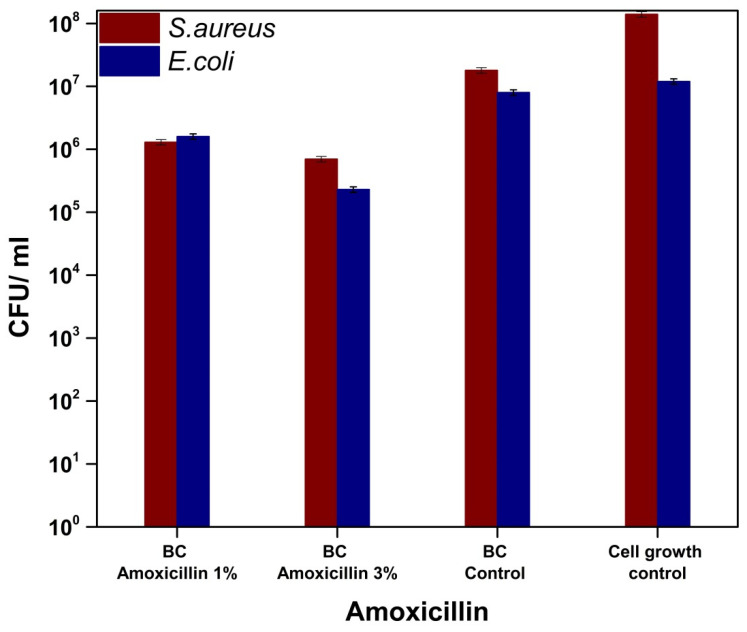
Graphical representation of CFU/mL values of *Staphylococcus aureus* ATCC 25923 and *Escherichia coli* ATCC 25922 in order to evaluate the capability of the bacterial cells to adhere on the surface of BC-amoxicillin samples.

**Figure 12:**
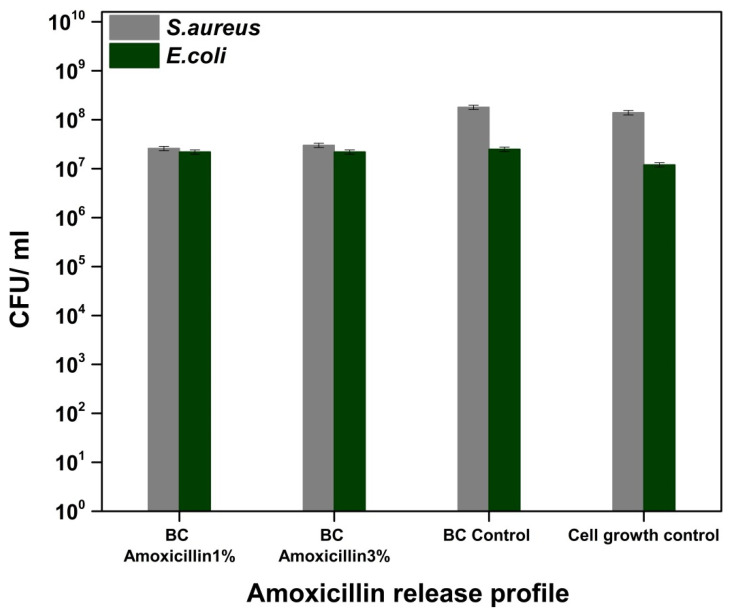
Graphical representation of CFU/mL values of *Staphylococcus aureus* ATCC 25923 and *Escherichia coli* ATCC 25922 in order to evaluate the amoxicillin release from the BC-Amoxicillin samples into the broth media.

Amoxicillin is a broad-spectrum antibiotic (Gram-positive bacteria, Gram-negative bacteria etc.) that belongs to the class of aminopenicillins, which are characterized by the presence of the β-lactam cycle and act by blocking the synthesis of the cell wall [47,48]. Figure 11 shows a slight decrease in CFU/mL values, respectively, and a weak ability to inhibit the adhesion of bacterial cultures on the surface of bacterial cellulose loaded with amoxicillin. However, we could not observe any significant change of the bacterial cell viability for *E. coli* and only a marginal antibacterial activity against *S. aureus* (about one order decrease of the viability), with this being correlated with the release of amoxicillin into the liquid medium (Figure 12).

Ye et al. [49] grafted/functionalized amoxicillin on bacterial cellulose to improve the antimicrobial activity of the biopolymer. The biological tests performed showed a significant improvement in antimicrobial activity and a non-toxic effect on HEK293 cells (*Human embryonic kidney 293 cells*), results that suggest the possibility of using them as wound dressings. Additionally, for this purpose, there are studies on bacterial cellulose loaded with different types of bacterial/antibiotic agents, such as amikacin [50], ceftriaxone [50,51], and tetracycline hydrochloride [52]. These results determine the possibility that the materials tested in this study (BC-bacitracin, BC-amoxicillin) can be used in the treatment of wounds as dressings.

## 3. Materials and Methods

### 3.1. Materials

Two antibiotics, bacitracin (from *Bacillus licheniformis*, ≥ 60,000 U/g (Potency)) and amoxicillin (≥ 900 µg/mg), produced by Sigma were used as received. During the whole experiments, distilled water was used.

### 3.2. Methods

#### 3.2.1. Synthesis of Bacterial Cellulose

The BC membranes were obtained in static culture using Hestrin-Schramm medium containing 3% fructose after 7 days. *Acetobacter* sp. strain used in this study was isolated from the traditionally fermented vinegar in Microbiology Laboratory of Chemical and Biochemical Engineering Department of Politehnica University of Bucharest. The obtained gel-like pellicles were purified by boiling in 0.1 N aqueous solution of NaOH for 1 h and rinsed with deionized water several times until the pH of the washing solution became neutral. The alkaline treatment could be considered as a standard procedure for removing bacteria and culture medium from BC gel membranes. This treatment can ensure a total loss of the viability of the bacterial cells. Biological impurities from the bacteria or from the fermentation medium could remained attached to the BC network but in very small quantities. The NaOH treatment is considered able to eradicate the bacteria by cell lysis. Concentrations of NaOH higher than 5% are not recommended because this could change the crystalline structure of BC from cellulose I to cellulose II [53,54].

#### 3.2.2. Synthesis of Bacitracin and Amoxicillin-Loaded BC Membranes

BC was lyophilized, loaded with active substances, and lyophilized again. The solutions of bacitracin and amoxicillin were made in two different concentrations of 1% and 3%, respectively. The freeze-dried BC samples with ~0.1 g weight was immersed in a volume of 5 mL of solution of amoxicillin/bacitracin, for 48 h, for both the absorption and adsorption of drugs. The antimicrobial agent concentrations (1% and 3%) were chosen to ensure antimicrobial activity, in order to evaluate the feasibility of the use of these drug delivery systems specially to prevent wound infection, not necessarily to treat the infection. Further works will be necessary to optimize the final compositions, taking care of the final applications and especially based on the in vivo assessments because the delivery rate is strongly correlated with the environment (not only pH but also the presence of exudate).

**Figure 13:**
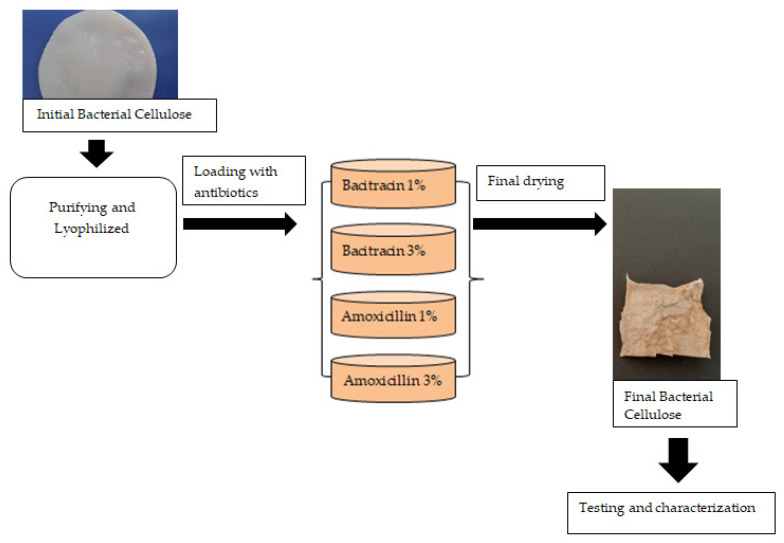
The technological process flowchart for manufacturing bacterial cellulose membranes loaded with bacitracin and amoxicillin.

In Figure 13, the obtained BC membranes’ technological flow loaded with different drugs is presented.

The obtained membranes were investigated by Fourier transform infrared spectroscopy (FTIR), thermal analysis (ATD-TG), and scanning electron microscopy (SEM). The kinetics of water absorption and in vitro antimicrobial activity against *Escherichia coli* and *Staphylococcus aureus* were also studied.

#### 3.2.3. FTIR Analysis

The synthesized products were characterized by FTIR using a Nicolet iS50FT-IR (Nicolet, City, MA, USA) spectrometer equipped with a DTGS detector, which provides information with a high sensitivity in the range of 4000 and 400 cm^−1^ at a resolution of 4 cm^−1^. All spectra were obtained by co-adding 32 scans, with the scanning time being 47 s.

#### 3.2.4. SEM Analysis

The SEM-EDS characterization was performed using a QUANTA INSPECT F50, FEI Company, Eindhoven, The Netherlands scanning electron microscope equipped with field emission gun electron-FEG (field emission gun) with 1.2 nm resolution and an energy dispersive X-ray spectrometer (EDS) with an MnK resolution of 133 eV.

#### 3.2.5. Thermal Analysis

The samples thermal analysis TG-DSC for the samples was performed with a Netzsch STA 449C Jupiter apparatus. Approximatively, 10 mg of each sample were placed in an open crucible made of alumina and heated with 10 K·min^−1^ from room temperature up to 900 °C, under the flow of 50 mL min^−1^ dried air. An empty alumina crucible was used as the reference.

#### 3.2.6. Water Absorption

The BC samples’ water absorption loaded with active substances was evaluated by determining the weight change of the samples during the adsorption in distilled water for 48 h. The measurements were made at different intervals of time: 0.25, 0.5, 1, 2, 4, 6, 24, and 48 h. The hydration capacity was calculated by measuring the initial weight (*M_i_*) and the weight of the sample after immersion in distilled water (*M_h,τ_*) for a defined period of time using Equation (1):

(1) $$Hydration\;capacity=\left(M_{h,\tau }-M_{i}\right)/M_{i}\times 100.$$

#### 3.2.7. Antibacterial Tests

The antibacterial activity was evaluated against *Staphylococcus aureus* ATCC 25923 and *Escherichia coli* ATCC 25922 by quantitatively determining the ability of selected strains to adhere to the surface of functionalized materials. The samples were placed in Petri dishes, with specimens of (1 cm x 1 cm). To avoid the impact of contaminants on the experiment, all specimens used were previously sterilized by maintaining UV radiation, 30 min on each side. In order to confirm the sterility of the tested samples before the antimicrobial assay, the sterility test was performed for each type of sample by maintaining it in nutrient broth media for 24 h, at 37 °C. The clarity of the broth media confirmed the sterility of the samples.

Microbial cell suspensions were made in sterile physiological buffer from cultures of 18–24 h with a standard density of 1.5 × 10^8^ CFU/mL. Each sample was incubated in liquid medium (nutrient broth ratio: bacterial suspension = 10:1) for 24 h at 37 °C; during which the bacterial cells multiplied in solution, and after reaching a density threshold, they adhered to the material surface. After this, each material fragment was extracted from the medium, gently washed, drop-wise, with 1 mL of sterile physiological buffer to remove non-adherent cells, and placed in sterile centrifuge tubes containing 1 mL of sterile physiological buffer. From the formed suspensions, 30 µL were taken and decimal dilutions were performed in order to quantitatively determine the number of bacterial cells, expressed in CFU/mL (colony forming units/mL). Serial decimal dilutions obtained from each sample, in sterile physiological buffer, were inoculated on the surface of the nutrient broth with 2% agar, in triplicate, and the number of viable cells was evaluated after incubation for 24 h at 37 °C, establishing the value of CFU/mL. The CFU/mL values were expressed as the average of the total number of colonies × 1/D (D = decimal dilution, for which the number of total colonies to determinate) [55,56].

At the same time, the release into the liquid medium of biologically active compounds was quantitatively evaluated based on the decimal microdilution method. After incubating the materials in a liquid medium in the presence of the 10^7^ bacterial cells density, 30 μL of the liquid media were taken and decimal dilutions were made in order to determine the number of viable bacterial cells, expressed in CFU/mL, according to the steps previously presented.

## 4. Conclusions

In this study, we aimed to investigate the possibility of using bio-cellulose as a potential antimicrobial wound dressing. Based on the obtained results, these samples are able to adsorb a large amount of water and exudates and also adsorb and release antibiotics (bacitracin and amoxicillin). The antimicrobial activity was induced by loading two antibiotics in the bacterial cellulose support, by adsorption. The grow rate of *Escherichia coli* and *Staphylococcus aureus* was lower in the presence of the bacterial cellulose loaded with antibiotics (bacitracin and amoxicillin) because of the release of these antimicrobial agents. According to these data, it can be concluded that the bacitracin- and amoxicillin-loaded bio-cellulose can be used as a potential antimicrobial wound dressing. The best results were obtained with bacitracin when both the antiadhesive and the adherence of the two bacterial strains as well as the antimicrobial activity in solution were promising. As future perspectives, the loading of the BC with different antibiotics (especially new-generation antibiotics) are desired in order to be tested as wound dressings and to continue with preclinical assessments, especially for the BC-bacitracin 3%, in order to evaluate the in vivo behavior and to optimize these formulations.

## Figures and Tables

**Table 1:** Assignment of relevant IR absorption bands of bacitracin, BC control, BC-bacitracin 1%, and BC-bacitracin 3%.

Assignment	Bacitracin Control Wavenumbers (cm^−1^)	BC Control Wavenumbers (cm^−1^)	BC-Bacitracin 1% Wavenumbers (cm^−1^)	BC-Bacitracin 3% Wavenumbers (cm^−1^)
C-O alcohol bond	1042	1056	1056	1055
C-O alcohol bond	1107	1108	1108	1107
asymmetric C-O-C stretch	1156	1160	1159	1160
CH_2_ symmetric bending	1308	1315	1315	1314
CH_2_ deformation	1455	1451	1455	1455
stretching vibrations of C-H and C-C	1522	1538	1521	1522
stretching vibrations of C-O	1643	1647	1648	1650
stretching vibrations of C-H and C-C	2961	2919	2957	2958

**Table 2:** Assignment of relevant IR absorption bands of amoxicillin.

Assignment	Amoxicillin Control Wavenumbers (cm^−1^)	BC Control Wavenumbers (cm^−1^)	BC-Amoxicillin 1% Wavenumbers (cm^−1^)	BC-Amoxicillin 3% Wavenumbers (cm^−1^)
νC = O (β-lactamic)	1772	1781	1771	1771
νC = O (amide)	1684	1647	1696	1696
C = C (aromatic)	1614	1631	1625	1625
νasCOO^−^	1576	1538	1556	1556
δNH (amide)	1517	1483	1520	1520
νsCOO^−^	1377	1368	1372	1371

**Table 3:** Residual mass of samples.

Sample	Temperature (°C)	Mass Loss (%)	Residual Mass (%)
BC Control	<200	1.30	10.63
	200–375	70.20	
	375–900	18.06	
BC-Bacitracin 1%	<200	2.37	8.39
	200–375	59.28	
	375–900	29.95	
BC-Bacitracin 3%	<200	2.50	12.16
	200–375	57.22	
	375–900	28.22	
BC-Amoxicillin 1%	<200	2.45	9.31
	200–375	60.52	
	375–900	27.72	
BC-Amoxicillin 3%	<200	2.73	6.57
	200–375	61.41	
	375–900	29.41	
